# Characterization of rare germline variants in familial multiple myeloma

**DOI:** 10.1038/s41408-021-00422-6

**Published:** 2021-02-13

**Authors:** Calogerina Catalano, Nagarajan Paramasivam, Joanna Blocka, Sara Giangiobbe, Stefanie Huhn, Matthias Schlesner, Niels Weinhold, Rolf Sijmons, Mirjam de Jong, Christian Langer, Klaus-Dieter Preuss, Björn Nilsson, Brian Durie, Hartmut Goldschmidt, Obul Reddy Bandapalli, Kari Hemminki, Asta Försti

**Affiliations:** 1 Division of Molecular Genetic Epidemiology, German Cancer Research Center (DKFZ), Heidelberg, Germany; 2 Department of Internal Medicine V, University of Heidelberg, Heidelberg, Germany; 3 Computational Oncology, Molecular Diagnostics Program, National Center for Tumor Diseases (NCT), Heidelberg, Germany; 4 National Center for Tumor Diseases Heidelberg (NCT), Heidelberg, Germany; 5 Bioinformatics and Omics Data Analytics, German Cancer Research Center (DKFZ), Heidelberg, Germany; 6 University Medical Center Groningen, University of Groningen, Groningen, The Netherlands; 7 Kempten Clinic, Kempten, Germany; 8 José Carreras Center for Immuno and Gene Therapy, Department of Internal Medicine I, Saarland University Medical School, Homburg (Saar), Germany; 9 Hematology and Transfusion Medicine, Department of Laboratory Medicine, Lund University, Lund, Sweden; 10 Cedars Sinai Cancer Center, Los Angeles, CA USA; 11 Hopp Children’s Cancer Center (KiTZ), Heidelberg, Germany; 12 Division of Pediatric Neurooncology, German Cancer Research Center (DKFZ), German Cancer Consortium (DKTK), Heidelberg, Germany; 13 Division of Cancer Epidemiology, German Cancer Research Center (DKFZ), Heidelberg, Germany; 14 Faculty of Medicine and Biomedical Center in Pilsen, Charles University in Prague, Pilsen, Czech Republic

**Keywords:** Myeloma, Cancer prevention, Genetics research

Dear Editor,

Multiple myeloma (MM) is a malignancy of plasma cells, characterized by the presence of monoclonal immunoglobulin, known as M protein^1^. MM is preceded by monoclonal gammopathy of undetermined significance (MGUS) which is also a precursor of immunoglobulin light chain (AL) amyloidosis^1^. Previous studies have reported a 2- to 4-fold increased risk of MGUS or MM in first-degree relatives of MM or MGUS patients, suggesting the existence of inherited susceptibility^2,3^. For many years, high-risk germline predisposing genes have been lacking for MM. However, recent sequencing efforts have proposed a few novel candidates, most notably loss-of-function (LoF) variants in the tumor suppressor gene *DIS3* and in the histone demethylase gene *KDM1A*^4–6^, and others as recently reviewed in detail in Pertesi et al. ^7^. In addition to the suspected rare, high-penetrance variants, genome-wide association studies have identified over 20 common, low-penetrance variants associated with the risk of MM; these were estimated to account for about 15% of the familial MM risk^8^.

As the genetic basis of most MM families remains unexplained, our study aimed at identifying germline predisposition genes in familial MM from Germany, Sweden, and the Netherlands, through whole genome and exome sequencing^9^. Altogether, 21 families with 46 affected and 20 unaffected family members were recruited (Supplementary Information, Supplementary Fig. 1). Each family had at least two individuals diagnosed with MM or its precursors MGUS and smoldering MM (SMM). After sequencing, detailed bioinformatics analyses using our in-house developed Familial Cancer Variant Prioritization Pipeline version-2 (FCVPPv2) were conducted to prioritize the most likely candidates (Fig. 1 and Supplementary Methods). Gradual filtering of variants after sequencing is shown for each family in Supplementary Table 1. The functions of the gene products were collected from the UniProtKB database (https://www.uniprot.org/) and literature search. They are summarized in Fig. 2 and details are shown in Supplementary Information.

**Fig. 1:**
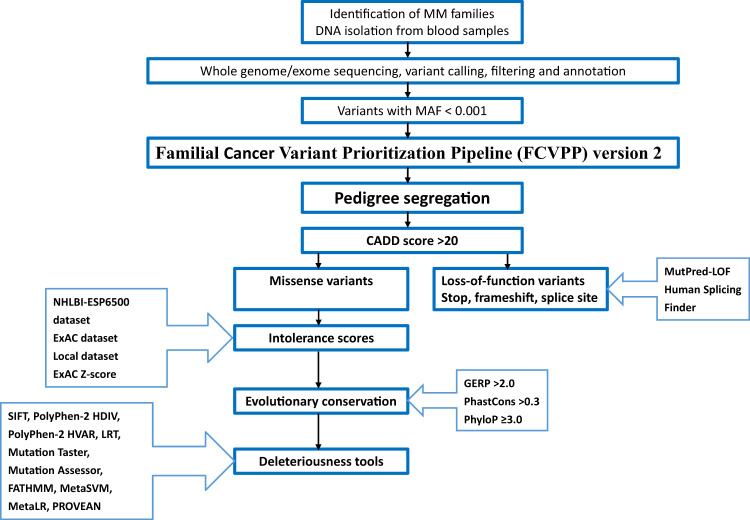
Pipeline for identification of missense and loss-of-function variants in the multiple myeloma families. After identification of the families, DNA isolation from the blood samples and whole genome or exome sequencing, variant calling, filtering, and annotation, we used our in-house developed Familial Cancer Variant Prioritization Pipeline v.2 to identify the most likely cancer predisposition variants for multiple myeloma. All variants with minor allele frequency (MAF) < 0.001 that segregated with the disease in the families were filtered by CADD score >20, which indicates the top 1% of potentially deleterious variants in the human genome. For missense variants, the corresponding genes were screened for their intolerance against functional variants using the NHLBI-ESP6500, ExAc, and local data sets as well the ExAC Z-score. The location of the variants was checked for evolutionary conservation using GERP (>2.0), PhastCons (>0.3), and PhyloP (≥3.0). Ten tools were used to predict the deleteriousness of the variants: Sorting Intolerant from Tolerant (SIFT), Polymorphism Phenotyping version-2 (PolyPhen-2) HDIV (HumDiv), PolyPhen-v2 HVAR (HumVar), Log ratio test (LRT), MutationTaster, Mutation Assessor, Functional Analysis Through Hidden Markov Models (FATHMM), MetaSVM, MetaLR, and Protein Variation Effect Analyzer (PROVEAN). For loss-of-function variants (frameshift and stopgain), pathogenic and neutral variants were predicted using MutPred-LOF with a threshold score of 0.50 at a 5% false-positive rate. Human Splicing Finder was used to evaluate the effect of splice site variants, with a yes/no score.

**Fig. 2:**
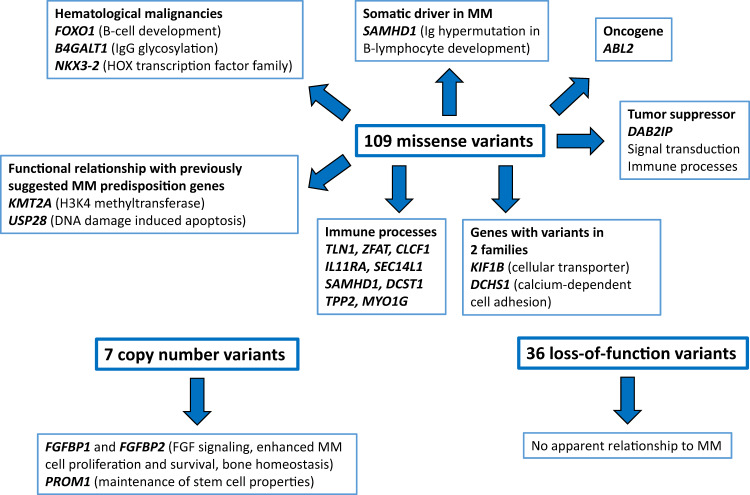
Summary of the identified variants in the multiple myeloma families. Total number of missense, loss-of-function, and copy-number variants are shown, and for the most intersting variants the function of the corresponding genes.

We identified 109 potential pathogenic missense variants; in most families, several candidates were found, and in four families none (Supplementary Table 2). All variants were private for each family, except for genes *KIF1B* and *DCHS1*, in which two different missense variants were found in two unrelated families (families 10 and 18 for *KIF1B* and 15 and 17 for *DCHS1*). KIF1B is involved in the transport of mitochondria and synaptic vesicles and DCHS1 is a calcium-dependent cell adhesion protein. Among the other genes harboring missense variants, tumor suppressor function was indicated for DAB2IP and oncogene function for ABL2. The former had diverse signal transduction functions and it is implicated in immune processes, as are TLN1, ZFAT, CLCF1, IL11RA, SEC14L1, SAMHD1, DCST1, TPP2, and MYO1G.

Another group of genes with key regulatory functions constituted *FOXO1, B4GALT1*, and *NKX3-2*. Transcription factor FOXO1 is a protein, which is the main target of insulin signaling, it increases osteoblast numbers and regulates B cell development. B4GALT1 is involved in the glycosylation of immunoglobulin G (IgG) and variants in this gene have been associated with IgG levels and hematological neoplasms, including MM. NKX3-2 (homeobox protein Nkx-3.2) is a member of the HOX gene transcription factors family, which are frequently dysregulated in hematologic malignancies.

Our candidate list included two genes, *KMT2A* and *USP28*, functionally related to the recently reported MM predisposing genes, *LSD1/KDM1A*, encoding a lysine-specific demethylase, and *USP45*, an apoptosis-related gene-regulating DNA repair^5,6^. KMT2A (alias MLL1) is a histone H3 lysine 4 (H3K4) methyltransferase, which plays an essential role in early development and hematopoiesis and which mediates chromatin modifications associated with epigenetic transcriptional activation. USP28 is a deubiquitinase involved in DNA damage-induced apoptosis. It regulates MYC protein stability in response to DNA damage.

We checked our gene list also for the presence of the 82 somatically mutated driver genes in MM, described in Walker et al. ^10^ and Maura et al. ^11^, but only *SAMHD1* passed all our in-house pipeline filters. SAMHD1 is a somatic driver in MM and the protein plays a role in maintaining dNTP levels in regulating DNA replication and damage repair. It enhances immunoglobulin hypermutation in B-lymphocyte development.

We also identified 36 loss-of-function (LoF) variants in the MM families (Supplementary Table 3). If we would apply a MutPred-LOF (http://mutpredlof.cs.indiana.edu/index.html) score higher than 0.50 at a 5% false-positive rate, only two frameshift variants, in the genes *SLC30A5* and *LONP2*, and six stop codon variants would pass the threshold. None of these had an apparent relationship to MM. Of the eight splice site variants, five were predicted by Human Splicing Finder (http://www.umd.be/HSF/HSF.shtml) to alter the splicing motifs (indicated by “yes” in Supplementary Table 3), however with no link to MM. Many of the genes with LoF mutations encode proteins with housekeeping functions, including LONP2, CSGALNACT2, HMGCLL1, and FUK.

We identified seven copy-number variants (CNVs) that segregated with MM in the families (Supplementary Table 4). These CNVs affected the coding regions of 11 genes. Duplication of chr4:15936942-16178663 in Family 5 covered the genes encoding fibroblast growth factor binding proteins FGFBP1 and FGFBP2, prominin 1 (*PROM1*), and transmembrane anterior posterior transformation protein 1 homolog (*TAPT1*). One of the primary genetic events in MM is t(4:14) translocation, creating a fusion between the immunoglobulin heavy chain (*IGH)* enhancer and *FGFR3* and leading to overexpression of *FGFR3*^12^*. FGFBP1* and *FGFBP2* encode proteins that are involved in FGF ligand bioactivation by releasing them from the extracellular matrix. Thus, duplication of these two genes may lead to activation of the FGF signaling, enhanced MM cell proliferation and survival, and affect bone homeostasis. PROM1 is involved in the suppression of cell differentiation and maintenance of stem cell properties.

All the identified variants were rare (allele frequency < 0.001) in the gnomAD database (https://gnomad.broadinstitute.org) and none of them was found as a germline variant in any cancer patient. However, COSMIC (https://cancer.sanger.ac.uk/cosmic) reported some of the variants as rare somatic mutations, mainly in cancer entities with a high mutation load, such as malignant melanoma and adenocarcinoma of the large intestine, but not in any hematological malignancies.

In a review of cancer-predisposing genes, it was observed that over 40% of germline variants were in genes that functioned also as somatic drivers^13^. In the above, we referred to some somatic drivers, and some of the observed genes are known to interact with key signaling pathways in MM, including PI3K/Akt/mTOR, Ras/Raf/MEK/MAPK, JAK/STAT, NF-κB, Wnt/β-catenin, and RANK/RANKL/OPG^14^. Among the relevant genes in our list, *DAB2IP*, encoding a Ras-GTPase activating protein, modulates key oncogenic pathways such as PI3K/Akt, NF-κB, and Wnt/β-catenin; *FOXO1* encodes for a downstream effector of Akt signaling; the *LRP1B* gene product negatively regulates the Wnt/β-catenin/TCF signaling, through its interaction with DVL2.

A somewhat surprising finding was that none of the 158 candidate genes matched with the genes linked to the 23 common, low-risk MM variants^8^. It is true that an overwhelming number of the published MM-related low-risk variants were located in the non-protein-coding region and some were distant from the coding regions^8^. The associated relative risks were mostly below 1.5, which would not be compatible with strong familial clustering. However, as many of the present families included only two affected members, chance clustering cannot be excluded. A further factor is that by the inclusion of persons with MGUS, which is more than one order of magnitude more prevalent than MM, certain genetic heterogeneity was introduced, in spite of the known shared genetic background^3^. Of note, the candidates that passed the pipeline included two genes, KMT2A and USP28, functionally related to the recently proposed high/moderate penetrance MM predisposing genes, *LSD1/KDM1A*, *USP45, ARID1A*, and *DIS3*^4–6^.

In few families, no candidate variants were found using the present criteria. Some possible reasons were explained in the above paragraph, and there are more possible reasons. In families of two affected individuals, polygenic risks would be more likely than in multiplex families of many affected individuals. In a previous study, evidence of enrichment of the common MM risk alleles among familial cases compared to sporadic cases or population-based controls was reported^15^. Our search did not consider polygenic risk. Even though the controls were tested for the presence of the M protein, a negative result suggests that the person would remain disease free only the next decade or two^9^. Finally, the present bioinformatics analysis was limited to coding variants.

In conclusion, we report here curated sequencing results from 21 MM/MGUS families. While most of the 154 presented candidate genes are unlikely to have a causal relationship to MM, the identified genes could be a valuable contribution to forthcoming, pooled sequencing efforts. Familial clustering of MM is rare and the set of 21 families was only possible through multicenter efforts. Based on the functional characterization and the literature review the strong candidates included *DAB2IP, ABL2, SAMHD1*, *KMT2A*, *USP28*, *FOXO1, B4GALT1*, *NKX3-2*, several immune-related genes, and *FGFBP1*, *FGFBP2*, and *PROM1* within the CNV in chromosome 4. Interestingly, many of these are somatic driver genes in cancer.

## Supplementary information

Supplementary Information

## Data Availability

Whole-genome sequencing data sets have been deposited to the European Genomephenome Archive (EGA) with accession number EGAS00001004734.
