# Supplementary material for: Characterization of rare germline variants in familial multiple myeloma
Source: Blood Cancer J. 2021 Feb 13;11(2):33. doi: 10.1038/s41408-021-00422-6 (PMC7882594; doi:10.1038/s41408-021-00422-6)
Supplement: Supplementary file 1 — Supplementary Information [file 41408_2021_422_MOESM1_ESM.pdf]

Supplementary Information

Characterization of rare germline variants in familial multiple myeloma

Calogerina Catalano[1,2,#], Nagarajan Paramasivam[3,#], Joanna Blocka[2], Sara Giangiobbe[1,2], Stefanie Huhn[2,4], Matthias Schlesner[5], Niels Weinhold[2], Rolf Sijmons[6], Mirjam de Jong[6], Christian Langer[7], Klaus-Dieter Preuss[8], Björn Nilsson[9], Brian Durie[10], Hartmut Goldschmidt[2,4], Obul Reddy Bandapalli[1,11,12,§], Kari Hemminki[1,13,14,§] and Asta Försti[1,11,12,§,*]

[1] Division of Molecular Genetic Epidemiology, German Cancer Research Center (DKFZ), Heidelberg, Germany
[2] Department of Internal Medicine V, University of Heidelberg, Heidelberg, Germany
[3] Computational Oncology, Molecular Diagnostics Program, National Center for Tumor Diseases (NCT), Heidelberg, Germany
[4] National Center for Tumor Diseases Heidelberg (NCT), Heidelberg, Germany
[5] Bioinformatics and Omics Data Analytics, German Cancer Research Center (DKFZ), Heidelberg, Germany
[6] University Medical Center Groningen, University of Groningen, Groningen, the Netherlands
[7] Kempten Clinic, Kempten, Germany
[8] José Carreras Center for Immuno and Gene Therapy, Department of Internal Medicine I, Saarland University Medical School, Homburg (Saar), Germany
[9] Hematology and Transfusion Medicine, Department of Laboratory Medicine, Lund University, Lund, Sweden
[10] Cedars Sinai Cancer Center, Los Angeles, CA, USA
[11] Hopp Children's Cancer Center (KiTZ), Heidelberg, Germany
[12] Division of Pediatric Neurooncology, German Cancer Research Center (DKFZ), German Cancer Consortium (DKTK), Heidelberg, Germany
[13] Division of Cancer Epidemiology, German Cancer Research Center (DKFZ), Heidelberg, Germany
[14] Faculty of Medicine and Biomedical Center in Pilsen, Charles University in Prague, Pilsen, Czech Republic

[#] Equal contribution
[§] Shared senior authorship
***** Correspondence to: Asta Försti, Division of Pediatric Neurooncology (B062), German Cancer Research Center (DKFZ), Im Neuenheimer Feld 580, 69120 Heidelberg, Germany, Tel.: +49-6221-421792, Fax: +49-6221-424639, Email: a.foersti@kitz-heidelberg.de

Materials and methods

Ethical approval

Collection of patient samples and associated clinico-pathological information was undertaken with written informed consent and relevant ethical review board approval at respective study centers in accordance with the tenets of the Declaration of Helsinki. The study was approved by the ethics committee of the Medical Faculty of the University of Heidelberg (study number S-589/2016), the Lund University Ethics Review Board (dnr 2010/131 and 2013/54) and the ethics committee of the Ärztekammer des Saarlandes (#154/08, #177/08). All patients enrolled by the University Medical Center Groningen (UMCG) were part of the Groningen-Heidelberg-Stettin EU TRANSCAN Familial cancer whole genome sequencing project. They were referred to UMCG clinically for diagnostics and counselling because of their cancer family history. Enrollment was therefore regarded as being directly in line with the clinical reason for testing and not subject to review by Ethics review board of the UMCG. All UMCG participants did sign informed consents stating that they agreed to undergo whole genome sequencing in this project to identify the cause of cancer predisposition (if any) in their families.

Multiple myeloma families

Altogether, 21 families with 46 affected and 20 unaffected family members were recruited (**Supplementary Figure 1**). Fifteen of the families were recruited in Germany, 12 in Heidelberg (1), two in Homburg and one in Ulm. Four families came from Sweden (2) and two from the Netherlands. Each family had at least two individuals diagnosed with MM or its precursors MGUS and smoldering MM (SMM). Also, patients with solitary plasmacytoma and AL amyloidosis were enrolled. Participating unaffected family members recruited in Heidelberg were analyzed for the

following parameters: blood count, creatinine, and glomerular filtration rate, calcium, immunoglobulin levels, free light chains and their ratio, protein electrophoresis, and immunofixation in serum and urine in order to exclude undetected MM or its precursor stages (1). Only individuals with negative immunofixation in serum and urine were considered as unaffected. The pedigrees of the German and Dutch families are shown in **Supplementary Figure 1**, each of the four Swedish families consisted of two first- or second-degree relatives diagnosed with MM.

Whole Genome Sequencing

Samples from Heidelberg and the Netherlands were whole-genome sequenced at the core facility of DKFZ. WGS of the MM family members was carried out using the Illumina X10 platform on DNA isolated from the peripherical blood samples (QIAamp® DNA Mini Kit. Qiagen). WGS was performed as paired-end sequencing with a read length of 150 bp. BWA mem (version 0.7.15, with parameters: -T 0) and Sambamba (version 0.6.5, with parameters: t 1 -l 0 --hash-table-size=2000000 --overflow-list-size=1000000 --io-buffer-size=64) were used to map sequences to the reference human genome (build GRC37, assembly hs37d5) and to remove duplicates, respectively. Platypus (version 0.8.1) was used for variant calling of small variants, single nucleotide variants (SNVs) and indels.

Whole-exome sequencing

Samples from Homburg, Ulm and Sweden were whole-exome sequenced as described in Halvarsson et. al. (2). Following isolation of exonic DNA by hybrid capture (SureSelect; Agilent, Santa Clara, CA), libraries were constructed using standard methods, and sequenced on Illumina HiSeq instruments to target mean coverage of 100x. Reads were aligned to GRCh37 and the resulting SAM files were converted to BAM files using Picard (http://picard.sourceforge.net). The

variants were called using multi sample processing mode of the Unified Genotyper tool from GATK. To calculate background frequencies, we used pre-existing WES data from the NHLBI GO Exome Sequencing Project, which were subjected to variant calling in the same run (3). Genotypes with quality <10 or read depth <8 were marked as missing data. Variants with >10% missing data were excluded, as were samples with >5% missing data. A total of 3,597 controls of European ancestry were selected from the ESP data using the first two principal components calculated using –mds-plot option in PLINK (4).

Variant annotation and filtering

The processed list of WES variants and raw WGS variants were analyzed together in the following downstream steps. The variants were annotated with Gencode v19 gene definitions using ANNOVAR (5) and further with dbSNP (6), 1000 Genomes phase III (7), dbNSFP v2.9 (8), and ExAC (9) read depth >10. Minor allele frequency (MAF) of 0.1% was used with respect to 1000 Genomes phase III and non-TCGA exome aggregation (ExAC, version 0.3) data to remove common variants, and variant frequency of 2% from the local data sets was used to remove technical artefacts. In order to control for family relatedness and sample swaps, a pairwise comparison of variants among the cohort was carried out.

Variant prioritization: missense variants

Variant prioritization was performed using our in-house developed pedigree-based pipeline, Familial Cancer Variant Prioritization Pipeline (FCVPP) version 2 (10). Pedigree segregation was the first criterion the variants were screened for. Family members diagnosed with MM, MGUS or AL amyloidosis were considered as cases, also members with plasma cell dyscrasia, solitary plasmacytoma and aberrant plasma cell clone were considered as variant carriers and unaffected

members as non-carriers, unless they were more than 10 years younger than the earliest age of diagnosis of cases in the family. After the pedigree segregation filtering, all the variants ranking within the top 1% of potentially deleterious variants in the human genome were selected using the Combined Annotation Dependent Depletion (CADD) tool v1.3; a scaled PHRED-like CADD score greater than 20 was applied (11).

As the following step, based on the assumption that variants within genes intolerant to variation are likely to be deleterious, only variants located in genes predicted to be intolerant by at least two of the Residual Variation Intolerance Scores (RVISs) based on NHLBI-ESP6500 (12) and ExAC (9) datasets and a local dataset, were selected. Additionally, they should be located in genes intolerant for missense variants according to Z-score, developed by the ExAC consortium (9).

As next, the variants should locate at an evolutionary conserved position, which was evaluated by Genomic Evolutionary Rate Profiling (GERP >2.0) (13), PhastCons (>0.3) (14) and Phylogenetic P-value (PhyloP ≥3.0) (15) with an inclusion cutoff of at least two positive predictions.

The variants were further screened for their potential deleteriousness by using 10 different prediction tools: Sorting Intolerant from Tolerant (SIFT) (16), Polymorphism Phenotyping version 2 (PolyPhen-2) HDIV (HumDiv) (17), PolyPhen-v2 HVAR (HumVar) (17), Log ratio test (LRT) (18), MutationTaster (19), Mutation Assessor (20), Functional Analysis Through Hidden Markov Models (FATHMM) (21), MetaSVM (8), MetaLR (8) and Protein Variation Effect Analyzer (PROVEAN) (22). Variants predicted to be deleterious by at least 60% of these tools were selected for further analyses.

Loss-of-function variant analysis

Frameshift, stop-gain/loss and splice-site variants affecting the canonical splice sites were considered if pedigree segregation and CADD score criteria were met. It is well known that also healthy people carry genetic variants predicted to cause loss-of function (LoF) (23). In order to discriminate pathogenic and neutral variants, we used MutPred-LOF (http://mutpredlof.cs.indiana.edu/index.html) (24). For each variant, it returns a score between zero and one; higher scores denote variants that are more likely to be pathogenic. In our analysis a threshold score of 0.50 at 5% false positive rate was used as suggested by Pagel et. al. (24). In addition, it shows up to five structural and functional mechanisms that are impacted in the affected region of the protein, accompanied by significant prior-corrected P-values. Variants that passed the filtering were further analyzed using the Translate tool (https://web.expasy.org/translate/) to translate a nucleotide (DNA/RNA) sequence to a protein sequence and IntOGen/c-BioPortal (https://www.intogen.org/search) in order to visualize the domain affected by the variant and the portion of the protein lost after the newly formed stop codon. Splice site variants were analyzed by using Human Splicing Finder (http://www.umd.be/HSF/HSF.shtml), a tool used to predict the effects of variants on splicing signals (25).

Additional variant quality control

Using the Integrative Genomics Viewer (IGV; version 2.4.10) (26), WGS data of all cases and controls were visually checked for correctness in order to increase the confidence of variant calls and reduce the risk of false positives.

Germline Copy Number Variant (gCNV) analysis

GATK gCNV module (version 4.1.7.0) was used to call CNVs from the WGS samples individually against a background of 200 WGS samples sequenced from the sample platform. The gCNVs were

called based on the best practice recommended by the GATK (https://gatk.broadinstitute.org/hc/en-us/articles/360035531152--How-to-Call-common-and-rare-germline-copy-number-variants). The major deviation was that the gCNVs were called only on the Gencode v19 exonic regions by considering them as the target regions. This decreased the turnaround time for the analysis of gCNVs from WGS data.

The resulting CNV segments with QS score above 30 were selected and annotated with the subset of gnomAD structural variant (SV) data (version 2.1, variants with 'PASS' filter tags and 'DUP' or 'DEL' SV types) using vcfanno (27). The segments with at least 80% overlap with a common gnomAD SV (popmax MAF > 0.1%) of same SV subtype were considered as common and removed. In addition, at least 50% of the targets (exons here) in the gCNV segments should have the denoised ploidies among the bottom (in the case on deletion) or top (in the case of duplication) 5% denoised cohort ploidies to be considered as a rare gCNVs. Subsequently, the candidate rare gCNVs were selected if they followed the disease inheritance pattern in the family.

Protein function

We used the UniProt Knowledgebase (UniProtKB, https://www.uniprot.org/) to evaluate the general function of the proteins, whose sequence was affected by the variants identified in our study (28).

Details of identified candidate genes, proteins and their function

In the main text we referred to some genes and gene variants and here we give functional details with references. The functions of the gene products were collected from the UniProtKB database and literature search.

Missense variants: genes, proteins and their function

After the FCVPPv2 application, a total of 109 potential pathogenic missense variants were identified; in most families several candidates were found and in four families none **(Supplementary Table 2)**. All variants were private for each family, except for two genes, *KIF1B (kinesin family member 1B)* and *DCHS1 (dachsous cadherin-related 1)*, in which two different missense variants were found in two unrelated families (Families 10 and 18 for *KIF1B* and 15 and 17 for *DCHS1*). KIF1B is involved in the transport of mitochondria and synaptic vesicles (29, 30). In Family 18, the variant (ENST00000263934, p.Asn1594Lys) was located between a domain of unknown function (DUF 3694) and the pleckstrin homology (PH) domain, which plays a role in recruiting proteins to different membranes and targeting them to appropriate cellular compartments. In Family 10, the variant (p.Leu181Met) was located within the kinesin motor domain. DCHS1 is a calcium-dependent cell adhesion protein. Both variants in *DCHS1* (ENST00000299441, p.Arg112Gln, p.Ser667Cys) were located within one of the extracellular cadherin domains, which are thought to mediate cell-cell contacts (CADD scores were 33 and 25).

Among the other genes harboring missense variants, *DAB2IP* has tumor suppressor but also oncogenic properties in many solid tumors and *ABL2* is an oncogene in T-cell acute lymphocytic leukemia and acute myeloid leukemia (31-33). The former has diverse signal transduction functions and it is implicated in immune processes, as are TLN1, ZFAT, CLCF1, IL11RA, SEC14L1, SAMHD1, DCST1, TPP2 and MYO1G. ZFAT and TPP2 are associated with autoimmune manifestations, and TPP2 and DCST1 with antigen presentation to T-cells; DCST1 additionally regulates type I interferon mediated innate immune response to control virus infection (34). TPP2 has been suggested to be the autoantigen target of MM and MGUS M-proteins (35).

Another group of genes with key regulatory functions constituted *FOXO1, B4GALT1*, and *NKX3-2*. FOXO1 is a member of the forkhead box family of transcription factors. It is the main target of insulin signaling and it increases osteoblast numbers and regulates B cell development (36). The protein interacts with recombination activating proteins (RAG1 and RAG2) that introduces DNA breaks at immunoglobulin genes required for V(D)J recombination in developing lymphocytes (37). FOXO1 mutations may thus contribute to aberrant RAG-dependent chromosomal translocations.

Other potentially relevant pathways include signal transduction (kinases and phosphatases), chromatin remodeling, hematopoiesis and apoptotic pathways, represented by a number of candidate genes, such as *B4GALT1*, *NKX3-2, KMT2A* and *USP28*. Glycosylation of immunoglobulin G (IgG) influences IgG effector functions and the addition of galactose to IgG glycans is synthesized by beta-1,4-galactosyltransferase 1, encoded by the *B4GALT1* gene. Variants in this gene were associated with IgG glycosylation levels, which correlated with some autoimmune diseases and hematological neoplasms, including MM (38, 39). NKX3-2 (homeobox protein Nkx-3.2) is a member of the HOX gene transcription factors family, which are frequently dysregulated in hematologic malignancies (40). Nkx-3.2 regulates expression of chondromodulin-1 in developing cartilage and in endochondral ossification (41).

Our candidate list included two genes, *KMT2A* and *USP28*, functionally related to the recently reported MM predisposing genes, *LSD1/KDM1A*, encoding a lysine-specific demethylase, and *USP45*, an apoptosis-related gene regulating DNA repair (42, 43). KMT2A (alias MLL1) is a histone H3 lysine 4 (H3K4) methyltransferase, which plays an essential role in early development and hematopoiesis and which mediates chromatin modifications associated with epigenetic transcriptional activation (44). Somatic mutations in KMT2 gene family are reported to be among the most frequent variants in many types of cancers, including MM (45). Carcinogenic mechanism

for KMT2A mutations and the common fusion genes, which KMT2A is a part of, may be related to transcription of homeobox (HOX) target genes (45). USP28 is a deubiquitinase involved in the DNA damage-induced apoptosis. It regulates MYC protein stability in response to DNA damage (46). In MM, overexpression of MYC, mainly through complex chromosomal rearrangements, has been shown to promote myeloma cell survival and to lead to poor prognosis (47).

We checked our gene list also for the presence of the 82 somatically mutated driver genes in MM, described in Walker et al. (48) and Maura et.al. (49), but only *SAMHD1* passed all our in-house pipeline filters. SAMHD1 is a somatic driver in MM and the protein plays a role in maintaining dNTP levels in regulating DNA replication and damage repair and counteracting viral infections (50). It enhances immunoglobulin hypermutation in B-lymphocyte development. The present variant (ENST00000262878, Gly211Arg) maps within the histidine/aspartate domain, which possesses triphosphohydrolase activity through which SAMHD1 hydrolyzes dNTPs to deoxynucleosides (51).

LoF variants: genes, proteins and their function

A total of 36 LoF variants were identified in the MM families **(Supplementary Table 3)**. If we would apply a MutPred-LOF score higher than 0.50 at a 5% false positive rate, as suggested by Pagel et.al. (24), only two frameshift variants, in the genes *SLC30A5* and *LONP2*, and six stop codon variants would pass the threshold. None of these had an apparent relationship to MM. Variants in the two genes related to immune function, *IL3RA* and *IL17REL*, had a low MutPred score. This score is not applicable to splice site variants. Of the eight splice site variants, five were predicted by Human Splicing Finder to alter the splicing motifs (indicate by 'yes' in **Supplementary Table 3**), however with no link to MM. Many of the genes with LoF mutations

encode proteins with housekeeping functions. LONP2 is an ATP-dependent protease that plays a role in maintaining peroxisome homeostasis. CSGALNACT2 is a member of the chondroitin N-acetylgalactosaminyltransferase family. HMGCLL1 is a non-mitochondrial 3-hydroxymethyl-3-methylglutaryl-CoA lyase involved in ketogenesis. FUK catalyzes the utilization of free L-fucose in glycoprotein and glycolipid synthesis.

Copy number variants: genes, proteins and their function

We identified seven CNVs that segregated with MM in the families (**Supplementary Table 4**). These CNVs affected the coding regions of 11 genes. Duplication of chr4:15936942-16178663 in Family 5 covered the genes encoding fibroblast growth factor binding proteins FGFBP1 and FGFBP2, prominin 1 (*PROM1)* involved in suppression of cell differentiation and maintenance of stem cell properties and transmembrane anterior posterior transformation protein 1 homolog (*TAPT1)*. One of the primary genetic events in MM is t(4:14) translocation, creating a fusion between the immunoglobulin heavy chain (*IGH)* enhancer and *FGFR3* and leading to overexpression of *FGFR3* (52). *FGFBP1* and *FGFBP2* encode proteins that are involved in FGF ligand bioactivation by releasing them from extracellular matrix. Thus, duplication of these two genes may lead to activation of the FGF signaling, enhanced MM cell proliferation and survival and affect bone homeostasis (53, 54). *PROM1* is considered a marker of both hematopoietic progenitor and stem cells and cancer stem cells and it is overexpressed in acute lymphoblastic leukemia and many solid cancers contributing to the growth of the cancer cells (55, 56).

In a review of cancer predisposing genes it was observed that over 40% of germline variants were in genes that functioned also as somatic drivers (57). In the above, we referred to some somatic drivers, and some of the observed genes are known to interact with key signaling pathways in MM,

including PI3K/Akt/mTOR, Ras/Raf/MEK/MAPK, JAK/STAT, NF-κB, Wnt/β-catenin, and RANK/RANKL/OPG (58). Among the relevant genes in our list, *DAB2IP,* encoding a Ras-GTPase activating protein, modulates key oncogenic pathways such as PI3K/Akt, NF-κB, and Wnt/β-catenin (31); *FOXO1* encodes for a downstream effector of Akt signaling (36); the *LRP1B* gene product negatively regulates the Wnt/β-catenin/TCF signaling, through its interaction with DVL2 (59).

References

1. Blocka J. et al. Familial Cancer: How to Successfully Recruit Families for Germline Mutations Studies? Multiple Myeloma as an Example. *Clin Lymphoma Myeloma Leuk*. **19**, 635-644 e632 (2019).
2. Halvarsson B. M. et al. Direct evidence for a polygenic etiology in familial multiple myeloma. *Blood Adv*. **1**, 619-623 (2017).
3. Carson A. R. et al. Effective filtering strategies to improve data quality from population-based whole exome sequencing studies. *BMC Bioinformatics*. **15**, 125 (2014).
4. Chang C. C. et al. Second-generation PLINK: rising to the challenge of larger and richer datasets. *Gigascience*. **4**, 7 (2015).
5. Wang K. et al. ANNOVAR: functional annotation of genetic variants from high-throughput sequencing data. *Nucleic Acids Res*. **38**, e164 (2010).
6. Smigielski E. M. et al. dbSNP: a database of single nucleotide polymorphisms. *Nucleic Acids Res*. **28**, 352-355 (2000).
7. Genomes Project C. et al. A global reference for human genetic variation. *Nature*. **526**, 68-74 (2015).
8. Liu X. et al. dbNSFP v3.0: A One-Stop Database of Functional Predictions and Annotations for Human Nonsynonymous and Splice-Site SNVs. *Hum Mutat*. **37**, 235-241 (2016).
9. Lek M. et al. Analysis of protein-coding genetic variation in 60,706 humans. *Nature*. **536**, 285-291 (2016).
10. Kumar A. et al. Familial Cancer Variant Prioritization Pipeline version 2 (FCVPPv2) applied to a papillary thyroid cancer family. *Sci Rep*. **8**, 11635 (2018).
11. Kircher M. et al. A general framework for estimating the relative pathogenicity of human genetic variants. *Nat Genet*. **46**, 310-315 (2014).
12. Petrovski S. et al. Genic intolerance to functional variation and the interpretation of personal genomes. *PLoS Genet*. **9**, e1003709 (2013).
13. Cooper G. M. et al. Distribution and intensity of constraint in mammalian genomic sequence. *Genome Res*. **15**, 901-913 (2005).
14. Siepel A. et al. Evolutionarily conserved elements in vertebrate, insect, worm, and yeast genomes. *Genome Res*. **15**, 1034-1050 (2005).
15. Pollard K. S. et al. Detection of nonneutral substitution rates on mammalian phylogenies. *Genome Res*. **20**, 110-121 (2010).

16. Kumar P. et al. Predicting the effects of coding non-synonymous variants on protein function using the SIFT algorithm. *Nat Protoc*. **4**, 1073-1081 (2009).
17. Adzhubei I. et al. Predicting functional effect of human missense mutations using PolyPhen-2. *Curr Protoc Hum Genet*. **Chapter 7**, Unit7 20 (2013).
18. Chun S. et al. Identification of deleterious mutations within three human genomes. *Genome Res*. **19**, 1553-1561 (2009).
19. Schwarz J. M. et al. MutationTaster evaluates disease-causing potential of sequence alterations. *Nat Methods*. **7**, 575-576 (2010).
20. Reva B. et al. Predicting the functional impact of protein mutations: application to cancer genomics. *Nucleic Acids Res*. **39**, e118 (2011).
21. Shihab H. A. et al. Predicting the functional, molecular, and phenotypic consequences of amino acid substitutions using hidden Markov models. *Hum Mutat*. **34**, 57-65 (2013).
22. Choi Y. et al. Predicting the functional effect of amino acid substitutions and indels. *PLoS One*. **7**, e46688 (2012).
23. Kaiser V. B. et al. Homozygous loss-of-function variants in European cosmopolitan and isolate populations. *Hum Mol Genet*. **24**, 5464-5474 (2015).
24. Pagel K. A. et al. When loss-of-function is loss of function: assessing mutational signatures and impact of loss-of-function genetic variants. *Bioinformatics*. **33**, i389-i398 (2017).
25. Desmet F. O. et al. Human Splicing Finder: an online bioinformatics tool to predict splicing signals. *Nucleic Acids Res*. **37**, e67 (2009).
26. Thorvaldsdottir H. et al. Integrative Genomics Viewer (IGV): high-performance genomics data visualization and exploration. *Brief Bioinform*. **14**, 178-192 (2013).
27. Pedersen B. S. et al. Vcfanno: fast, flexible annotation of genetic variants. *Genome Biol*. **17**, 118 (2016).
28. UniProt C. UniProt: a worldwide hub of protein knowledge. *Nucleic Acids Res*. **47**, D506-D515 (2019).
29. Matsushita M. et al. A novel kinesin-like protein, KIF1Bbeta3 is involved in the movement of lysosomes to the cell periphery in non-neuronal cells. *Traffic*. **5**, 140-151 (2004).
30. Nangaku M. et al. KIF1B, a novel microtubule plus end-directed monomeric motor protein for transport of mitochondria. *Cell*. **79**, 1209-1220 (1994).
31. Bellazzo A. et al. Block one, unleash a hundred. Mechanisms of DAB2IP inactivation in cancer. *Cell Death Differ*. **24**, 15-25 (2017).
32. Greuber E. K. et al. Role of ABL family kinases in cancer: from leukaemia to solid tumours. *Nat Rev Cancer*. **13**, 559-571 (2013).
33. Liu L. et al. DAB2IP in cancer. *Oncotarget*. **7**, 3766-3776 (2016).
34. Nair S. et al. Global functional profiling of human ubiquitome identifies E3 ubiquitin ligase DCST1 as a novel negative regulator of Type-I interferon signaling. *Sci Rep*. **6**, 36179 (2016).
35. Preuss K. D. et al. Identification of antigenic targets of paraproteins by expression cloning does not support a causal role of chronic antigenic stimulation in the pathogenesis of multiple myeloma and MGUS. *Int J Cancer*. **121**, 459-461 (2007).
36. Ushmorov A. et al. FOXO in B-cell lymphopoiesis and B cell neoplasia. *Semin Cancer Biol*. **50**, 132-141 (2018).
37. Ochodnicka-Mackovicova K. et al. The DNA Damage Response Regulates RAG1/2 Expression in Pre-B Cells through ATM-FOXO1 Signaling. *J Immunol*. **197**, 2918-2929 (2016).
38. Lauc G. et al. Loci associated with N-glycosylation of human immunoglobulin G show pleiotropy with autoimmune diseases and haematological cancers. *PLoS Genet*. **9**, e1003225 (2013).

39. Zhang Z. et al. Serum protein N-glycosylation changes in multiple myeloma. *Biochim Biophys Acta Gen Subj*. **1863**, 960-970 (2019).
40. Agnelli L. et al. Overexpression of HOXB7 and homeobox genes characterizes multiple myeloma patients lacking the major primary immunoglobulin heavy chain locus translocations. *Am J Hematol*. **86**, E64-66 (2011).
41. Zhu S. et al. Chondromodulin-1 in health, osteoarthritis, cancer, and heart disease. *Cell Mol Life Sci*. **76**, 4493-4502 (2019).
42. Waller R. G. et al. Novel pedigree analysis implicates DNA repair and chromatin remodeling in multiple myeloma risk. *PLoS Genet*. **14**, e1007111 (2018).
43. Wei X. et al. Germline Lysine-Specific Demethylase 1 (LSD1/KDM1A) Mutations Confer Susceptibility to Multiple Myeloma. *Cancer Res*. **78**, 2747-2759 (2018).
44. Dupere-Richer D. et al. Epigenetic regulatory mutations and epigenetic therapy for multiple myeloma. *Curr Opin Hematol*. **24**, 336-344 (2017).
45. Fagan R. J. et al. COMPASS Ascending: Emerging clues regarding the roles of MLL3/KMT2C and MLL2/KMT2D proteins in cancer. *Cancer Lett*. **458**, 56-65 (2019).
46. Popov N. et al. The ubiquitin-specific protease USP28 is required for MYC stability. *Nat Cell Biol*. **9**, 765-774 (2007).
47. Walker B. A. et al. Translocations at 8q24 juxtapose MYC with genes that harbor superenhancers resulting in overexpression and poor prognosis in myeloma patients. *Blood Cancer J*. **4**, e191 (2014).
48. Walker B. A. et al. Identification of novel mutational drivers reveals oncogene dependencies in multiple myeloma. *Blood*. **132**, 587-597 (2018).
49. Maura F. et al. Genomic landscape and chronological reconstruction of driver events in multiple myeloma. *Nat Commun*. **10**, 3835 (2019).
50. Kohnken R. et al. Regulation of deoxynucleotide metabolism in cancer: novel mechanisms and therapeutic implications. *Mol Cancer*. **14**, 176 (2015).
51. Hartmann G. Nucleic Acid Immunity. *Adv Immunol*. **133**, 121-169 (2017).
52. Foltz S. M. et al. Evolution and structure of clinically relevant gene fusions in multiple myeloma. *Nat Commun*. **11**, 2666 (2020).
53. Krejci P. et al. The fibroblast growth factors in multiple myeloma. *Leukemia*. **20**, 1165-1168 (2006).
54. Labanca E. et al. Fibroblast growth factors signaling in bone metastasis. *Endocr Relat Cancer*. **27**, R255-R265 (2020).
55. Godfrey L. et al. H3K79me2/3 controls enhancer-promoter interactions and activation of the pan-cancer stem cell marker PROM1/CD133 in MLL-AF4 leukemia cells. *Leukemia*. (2020).
56. Saha S. K. et al. PROM1 and PROM2 expression differentially modulates clinical prognosis of cancer: a multiomics analysis. *Cancer Gene Ther*. **27**, 147-167 (2020).
57. Rahman N. Realizing the promise of cancer predisposition genes. *Nature*. **505**, 302-308 (2014).
58. Hu J. et al. Targeting signaling pathways in multiple myeloma: Pathogenesis and implication for treatments. *Cancer Lett*. **414**, 214-221 (2018).
59. Wang Z. et al. Down-regulation of LRP1B in colon cancer promoted the growth and migration of cancer cells. *Exp Cell Res*. **357**, 1-8 (2017).

Supplementary Table 1. Summary of the variants identified in the multiple myeloma families. Number of variants after each step of Familial Cancer Variant Prioritization Pipeline version 2 is shown for each family.

Family ID	No cases	No possible carriers	No healthy	MAF <0.1%	Pedigree segregation	CADD >20	Nonsynonymous variants	Frameshift /stopgain/ splice site variants	Non-coding variants	Nonsynonymous variants after pipeline	Frameshift/ stopgain variants after filtering	Splice site variants after filtering
Family 1	2		2	73103	8170	55	23	1	31	5	0	1
Family 2	2			38348	12921	88	44	1	43	14	0	1
Family 3	2		2	82691	1306	3	2	0	1	0	0	0
Family 4	2	2		75478	3072	6	3	0	3	0	0	0
Family 5	2		1	73995	1159	7	5	1	1	1	0	0
Family 6	2	1		106996	33089	187*	74	9	101	6	7	2
Family 7	2	2		72315	15397	101[#]	53	6	41	8	4	0
Family 8	4	2		91888	3496	13	5	1	7	0	0	1
Family 9	3			46742	7027	40[#]	21	2	16	6	0	0
Family 10	2		1	51782	7223	48[#]	28	3	16	4	2	1
Family 11	2			41753	12709	108[µ]	58	4	45	7	3	1
Family 12	2			49627	4815	28	14	0	14	3	0	0
Family 13**	2	2		938	208	51	46	5	0	12	5	0
Family 14**	2		2	862	20	4	4	0	0	0	0	0
Family 15**	2			626	197	46[§]	42	2	0	7	2	0
Family 16	2		1	50077	6775	47[#]	20	3	23	2	1	0
Family 17	3	1	1	73373	3014	18	12	1	5	3	1	0
Family 18**	2			462	135	33	30	2	1	5	0	0
Family 19**	2			497	163	35	34	1	0	8	0	1
Family 20**	2			496	171	46[µ]	37	6	2	10	5	1
Family 21**	2			477	142	35	33	1	1	8	1	0

MAF, minor allele frequency; CADD, Combined Annotation-Dependent Depletion

** Whole exome sequencing

* Includes 2 nonframeshift and 1 synonymous variants

[#] Includes 1 nonframeshift variant

[μ] Includes 1 synonymous variant

[§] Includes 2 synonymous variant

Supplementary Table 2. Missense variants prioritized using the FCVPPv2.

Family_ID	GENE	Gene name	CHROM_POS_REF_ALT	Ensembl transcript:exon:nucleotide: amino acid	CADD	ExAC z-score	Deleteriousness score (n/10)*	Function
**Family 1**	CNOT10	CCR-NOT Transcription Complex Subunit 10	3_32776360_T_C	ENST00000328834.5:exon12:c.T1406C:p.L469S	28.80	0.71	6	transcription; translation regulation
	PTPRG	Protein Tyrosine Phosphatase Receptor Type G	3_62268487_T_C	ENST00000474889.1:exon28:c.T3998C:p.I1333T	28.10	0.34	10	protein phosphatase
	BPGM	Bisphosphoglycerate Mutase	7_134346548_G_A	ENST00000393132.2:exon3:c.G289A:p.G97S	29.00	0.77	8	glycolysis
	SNX25	Sorting Nexin 25	4_186185615_T_C	ENST00000504273.1:exon4:c.T263C:p.F88S	28.00	0.07	7	protein transport
	TAB1	TGF-Beta Activated Kinase 1 (MAP3K7) Binding Protein 1	22_39814820_G_A	ENST00000216160.6:exon6:c.G634A:p.E212K	24.70	1.79	6	intracellular signaling
**Family 2**	NADSYN1	NAD Synthetase 1	11_71201935_T_G	ENST00000319023.2:exon17:c.T1607G:p.I536S	29.60	0.06	7	NAD biosynthesis/metabolism
	TCTN1	Tectonic Family Member 1	12_111057706_G_A	ENST00000397659.4:exon2:c.G286A:p.D96N	32.00	0.20	9	cilium biogenesis/degradation
	SLC8A3	Solute Carrier Family 8 Member A3	14_70633677_C_G	ENST00000381269.2:exon2:c.G1463C:p.R488P	25.60	0.00	6	Ca2+ homeostasis
	NEURL	Neuralized E3 Ubiquitin Protein Ligase 1	10_105344805_C_T	ENST00000369780.4:exon4:c.C1162T:p.R388C	34.00	3.48	7	ubiquitination; Notch signaling
	SEMA3F	Semaphorin 3F	3_50225153_C_A	ENST00000002829.3:exon19:c.C1963A:p.R655S	34.00	2.68	7	neuronal development; anti-tumorigenic in endothelial cells
	MYH7	Myosin Heavy Chain 7	14_23898252_A_G	ENST00000355349.3:exon14:c.T1319C:p.V440A	25.80	6.54	9	muscle myosin
	LRP1B	LDL Receptor Related Protein 1B	2_141473606_G_A	ENST00000389484.3:exon37:c.C5959T:p.R1987C	34.00	0.52	9	endocytosis
	EXOC3L4	Exocyst Complex Component 3 Like 4	14_103576417_C_T	ENST00000380069.3:exon11:c.C2026T:p.R676W	33.00	0.72	7	exocytosis
	ABCF2	ATP Binding Cassette Subfamily F Member 2	7_150923514_C_T	ENST00000287844.2:exon2:c.G31A:p.A11T	23.50	2.70	6	cellular transporter?
	DAB2IP	DAB2 Interacting Protein	9_124522455_G_A	ENST00000259371.2:exon6:c.G823A:p.G275R	29.00	2.61	9	tumor suppressor; signal transduction; immune response

	NKX3-2	NK3 Homeobox 2	4_13543940_G_T	ENST00000382438.5:exon2:c.C679A:p.H227N	28.20	1.00	9	skeletal development; transcription regulation
	TLN1	Talin 1	9_35725284_G_T	ENST00000314888.9:exon3:c.C165A:p.D55E	26.20	5.13	8	cell adhesion; bone metabolism; immune response
	MYH14	Myosin Heavy Chain 14	19_50752254_C_T	ENST00000425460.1:exon13:c.C1340T:p.A447V	34.00	1.70	9	cellular myosin
	RSPRY1	Ring Finger And SPRY Domain Containing 1	16_57250905_C_T	ENST00000537866.1:exon8:c.C859T:p.R287C	33.00	3.08	8	unknown function
**Family 5**	BRD3	Bromodomain Containing 3	9_136918535_G_C	ENST00000303407.7:exon2:c.C65G:p.P22R	23.70	3.71	7	chromatin remodeling
**Family 6**	TSNAX, TSNAX-DISC1	Translin Associated Factor X, TSNAX-DISC1 Readthrough (NMD Candidate)	1_231696901_C_G	ENST00000366639.4:exon5:c.C395G:p.S132C	28.40	.,.	9	differentiation; spermatogenesis
	UGDH	UDP-Glucose 6-Dehydrogenase	4_39523035_G_A	ENST00000316423.6:exon2:c.C98T:p.T33M	26.70	1.95	8	carbohydrate metabolism
	WNT7A	Wnt Family Member 7A	3_13916462_C_T	ENST00000285018.4:exon2:c.G280A:p.G94R	34.00	2.64	9	development; Wnt signaling
	RP4-583P15.1, ZGPAT	Zinc Finger CCCH-Type And G-Patch Domain Containing	20_62366093_G_T	ENST00000328969.5:exon5:c.G968T:p.S323I	28.00	.,.	7	transcription repressor
	ZFAT	Zinc Finger And AT-Hook Domain Containing	8_135490814_C_T	ENST00000377838.3:exon16:c.G3643A:p.G1215R	26.00	0.96	6	transcriptional regulator; immune response
	SEMA3G	Semaphorin 3G	3_52473704_C_T	ENST00000231721.2:exon12:c.G1459A:p.V487M	33.00	0.25	7	endothelial cell migration
**Family 7**	CLCF1	Cardiotrophin Like Cytokine Factor 1	11_67132822_C_T	ENST00000312438.7:exon3:c.G463A:p.G155S	27.90	0.81	8	neuronal development; immune response
	MGAT5B	Alpha-1,6-Mannosylglycoprotein 6-Beta-N-Acetylglucosaminyltransferase B	17_74942495_G_T	ENST00000569840.2:exon16:c.G1886T:p.G629V	31.00	2.24	8	protein modification/glycosylation
	MYRF	Myelin Regulatory Factor	11_61539096_C_G	ENST00000265460.5:exon6:c.C838G:p.P280A	23.50	2.39	6	oligodendrocyte differentiation
	HOXC8	Homeobox C8	12_54404885_G_A	ENST00000040584.4:exon2:c.G449A:p.R150H	29.60	1.44	10	cartilage differentiation
	DSP	Desmoplakin	6_7580073_C_T	ENST00000379802.3:exon23:c.C3650T:p.T1217M	24.40	0.91	6	desmosomal cell adhesion

	SLC22A6	Solute Carrier Family 22 Member 6	11_62744737_A_T	ENST00000377871.3:exon9:c.T1484A:p.V495E	24.70	0.04	7	organic anion transport
	B4GALT1	Beta-1,4-Galactosyltransferase 1	9_33166776_G_A	ENST00000379731.4:exon1:c.C392T:p.P131L	28.70	1.73	7	glycoconjugate and lactose biosynthesis; cell/matrix adhesion
	SEMA5A	Semaphorin 5A	5_9197323_C_A	ENST00000382496.5:exon10:c.G1025T:p.R342L	34.00	1.68	6	neurogenesis; angiogenesis
**Family 9**	FRY	FRY Microtubule Binding Protein	13_32698466_G_A	ENST00000380250.3:exon5:c.G508A:p.A170T	33.00	4.03	7	mitotic check-point
	PDE1A	Phosphodiesterase 1A	2_183070764_G_C	ENST00000435564.1:exon8:c.C853G:p.R285G	34.00	0.45	10	signal transduction
	FOXO1	Forkhead Box O1	13_41240315_G_T	ENST00000379561.5:exon1:c.C35A:p.P12Q	26.10	2.77	9	transcription factor; insulin signaling; bone metabolism
	WNK3	WNK Lysine Deficient Protein Kinase 3	X_54263446_G_C	ENST00000354646.2:exon20:c.C4553G:p.S1518C	26.80	1.57	7	electrolyte homeostasis
	IL11RA	Interleukin 11 Receptor Subunit Alpha	9_34658519_C_T	ENST00000555003.1:exon8:c.C649T:p.R217C	25.30	0.30	6	bone metabolism; immune response
	SCN4A	Sodium Voltage-Gated Channel Alpha Subunit 4	17_62018409_G_A	ENST00000435607.1:exon24:c.C5233T:p.R1745C	24.60	1.23	10	sodium channel
**Family 10**	KIF1B	Kinesin Family Member 1B	1_10428554_C_G	ENST00000263934.6:exon42:c.C4644G:p.N1548K	31.00	4.04	6	cellular transporter
	PHACTR4	Phosphatase And Actin Regulator 4	1_28800291_A_G	ENST00000373839.3:exon7:c.A1049G:p.H350R	22.60	0.56	6	neurogenesis
	SLC50A1	Solute Carrier Family 50 Member 1	1_155109327_G_A	ENST00000368404.4:exon3:c.G182A:p.G61E	28.50	1.13	10	sugar transporter
	BIRC6	Baculoviral IAP Repeat Containing 6	2_32626383_G_A	ENST00000421745.2:exon7:c.G1187A:p.C396Y	26.50	1.40	7	apoptosis; ubiquitination
**Family 11**	KIAA1199	CEMIP, Cell Migration Inducing Hyaluronidase 1	15_81201631_G_A	ENST00000394685.3:exon14:c.G1781A:p.G594D	29.00	2.18	10	epithelial-mesenchymal transition
	EPOR	Erythropoietin Receptor	19_11491590_A_T	ENST00000222139.6:exon6:c.T797A:p.L266Q	28.80	2.20	9	signal transduction
	ZNF236	Zinc Finger Protein 236	18_74589998_T_C	ENST00000253159.8:exon7:c.T868C:p.C290R	27.50	4.89	7	transcription regulation
	GRM4	Glutamate Metabotropic Receptor 3	6_34024438_G_A	ENST00000538487.2:exon6:c.C1051T:p.R351C	34.00	3.48	10	glutamatergic neurotransmission

	DHX16	DEAH-Box Helicase 16	6_30638221_C_T	ENST00000376442.3:exon4:c.G632A:p.R211H	27.50	3.68	7	mRNA processing; cell cycle progression
	FLNC	Filamin C	7_128492756_C_T	ENST00000325888.8:exon36:c.C5954T:p.S1985L	34.00	4.62	7	muscle-specific actin-cross-linking protein
	SYNJ1	Synaptojanin 1	21_34029139_C_T	ENST00000433931.2:exon21:c.G2770A:p.V924I	25.90	1.68	9	endocytosis
**Family 12**	TGIF2, TGIF2-C20orf24	TGFB Induced Factor Homeobox 2, TGIF2-RAB5IF Readthrough	20_35207278_G_A	ENST00000373874.2:exon2:c.G101A:p.R34Q	34.00	.,.	8	transciption repression
	AMPD3	Adenosine Monophosphate Deaminase 3	11_10516449_C_T	ENST00000396554.3:exon8:c.C1165T:p.R389W	35.00	1.02	10	erythrocyte homeostasis
	BTBD2	BTB Domain Containing 2	19_1987659_C_T	ENST00000255608.4:exon6:c.G1021A:p.E341K	35.00	2.93	7	protein-protein interaction
**Family 13**	TRIM71	Tripartite Motif Containing 71	3_32932492_A_G	ENST00000383763.5:exon4:c.A1796G:p.E599G	24.00	5.59	10	RNA-mediated gene silencing; ubiquitination
	EIF2B3	Eukaryotic Translation Initiation Factor 2B Subunit Gamma	1_45446770_G_A	ENST00000360403.2:exon2:c.C71T:p.P24L	32.00	0.56	10	protein biosynthesis
	BBS2	Bardet-Biedl Syndrome 2	16_56540081_T_C	ENST00000245157.5:exon6:c.A668G:p.N223S	23.70	0.72	9	cilium biogenesis/degradation; protein transport
	KMT2A	Lysine Methyltransferase 2A	11_118376242_C_T	ENST00000534358.1:exon27:c.C9635T:p.T3212I	23.20	6.64	6	histone modification; hematopoiesis
	DLGAP4	DLG Associated Protein 4	20_35060539_G_A	ENST00000373913.3:exon3:c.G419A:p.R140H	31.00	3.26	7	molecular organization of synapses; neuronal cell signaling
	INTS5	Integrator Complex Subunit 5	11_62415289_C_T	ENST00000330574.2:exon2:c.G2263A:p.G755S	29.60	2.28	7	snRNA transcription and processing
	HSPB1	Heat Shock Protein Family B (Small) Member 1	7_75932109_G_C	ENST00000248553.6:exon1:c.G80C:p.R27P	31.00	1.54	9	molecular chaperone
	TLE1	TLE Family Member 1, Transcriptional Corepressor	9_84228369_G_A	ENST00000376499.3:exon12:c.C986T:p.P329L	25.70	3.23	7	transcriptional corepressor
	SEC14L1	SEC14 Like Lipid Binding 1	17_75205479_A_G	ENST00000436233.4:exon14:c.A1532G:p.E511G	34.00	2.60	7	signal transduction inhibition; innate immunity
	THRB	Thyroid Hormone Receptor Beta	3_24185122_T_C	ENST00000396671.2:exon8:c.A608G:p.E203G	24.40	2.84	10	transcription regulation

	UFC1	Ubiquitin-Fold Modifier Conjugating Enzyme 1	1_161123804_C_T	ENST00000368003.5:exon1:c.C17T:p.T6M	32.00	0.54	7	ubiquitination
	STARD10	StAR Related Lipid Transfer Domain Containing 10	11_72466798_C_T	ENST00000334805.6:exon6:c.G578A:p.G193D	32.00	1.78	6	lipid transporter
**Family 15**	COL5A2	Collagen Type V Alpha 2 Chain	2_189957140_G_A	ENST00000374866.3:exon7:c.C463T:p.R155C	32.00	1.11	9	collagen
	SAMHD1	SAM And HD Domain Containing Deoxynucleoside Triphosphate Triphosphohydrolase 1	20_35555650_C_T	ENST00000262878.4:exon6:c.G631A:p.G211R	34.00	2.20	10	immune response
	DCHS1	Dachsous Cadherin-Related 1	11_6662510_C_T	ENST00000299441.3:exon2:c.G335A:p.R112Q	33.00	2.34	6	calsium-dependent cell adhesion
	KLHDC3	Kelch Domain Containing 3	6_42986424_C_T	ENST00000326974.4:exon7:c.C787T:p.R263W	35.00	3.83	7	meiotic recombination
	ATL3	Atlastin GTPase 3	11_63419412_A_C	ENST00000398868.3:exon5:c.T557G:p.L186R	29.20	0.82	10	endoplasmic reticulum tubular network biogenesis
	WFS1	Wolframin ER Transmembrane Glycoprotein	4_6296854_G_A	ENST00000226760.1:exon7:c.G799A:p.D267N	24.10	.	6	Ca2+ homeostasis
	FGG	Fibrinogen Gamma Chain	4_155533353_C_T	ENST00000336098.3:exon3:c.G124A:p.G42S	32.00	0.28	10	hemostasis
**Family 16**	DCST1	DC-STAMP Domain Containing 1	1_155006565_C_T	ENST00000295542.1:exon2:c.C53T:p.P18L	25.90	0.41	6	immune response
	S100A11	S100 Calcium Binding Protein A11	1_152006260_G_A	ENST00000271638.2:exon2:c.C20T:p.P7L	29.20	0.02	6	regulation of cellular processes
**Family 17**	HERC1	HECT And RLD Domain Containing E3 Ubiquitin Protein Ligase Family Member 1	15_64010835_G_A	ENST00000443617.2:exon21:c.C3916T:p.R1306C	33.00	3.83	6	antigen processing; protein ubiquitination
	DCHS1	Dachsous Cadherin-Related 1	11_6655238_G_C	ENST00000299441.3:exon4:c.C2000G:p.S667C	25.20	2.34	7	calcium-dependent cell adhesion
	SLC12A7	Solute Carrier Family 12 Member 7	5_1087125_A_G	ENST00000264930.5:exon6:c.T568C:p.S190P	24.80	1.65	10	ion transport
**Family 18**	KIF1B	Kinesin Family Member 1B	1_10327549_C_A	ENST00000263934.6:exon6:c.C541A:p.L181M	27.10	4.04	8	cellular transporter
	SLC2A4	Solute Carrier Family 2 Member 4	17_7188211_G_A	ENST00000317370.8:exon8:c.G973A:p.A325T	29.50	0.64	9	glucose transporter

	ATP11A	ATPase Phospholipid Transporting 11A	13_113512584_G_A	ENST00000487903.1:exon22:c.G2647A:p.V883M	24.50	1.44	10	lipid transporter
	ITPRIP	Inositol 1,4,5-Trisphosphate Receptor Interacting Protein	10_106074540_C_T	ENST00000278071.2:exon3:c.G1270A:p.G424S	31.00	0.39	6	intracellular calcium signaling
	GPR125	ADGRA3; Adhesion G Protein-Coupled Receptor A3	4_22390532_T_C	ENST00000334304.5:exon19:c.A2762G:p.Y921C	26.20	0.73	7	signal transduction
**Family 19**	ABL2	ABL Proto-Oncogene 2, Non-Receptor Tyrosine Kinase	1_179095661_G_A	ENST00000502732.1:exon4:c.C538T:p.R180C	34.00	1.31	10	cell growth and survival
	PDCD11	Programmed Cell Death 11	10_105173778_G_A	ENST00000369797.3:exon10:c.G1241A:p.G414E	26.00	0.56	7	rRNA processing
	TPP2	Tripeptidyl Peptidase 2	13_103299657_A_G	ENST00000376065.4:exon21:c.A2591G:p.K864R	28.90	3.07	9	immune response
	HHIPL1	HHIP Like 1	14_100129358_G_A	ENST00000330710.5:exon6:c.G1648A:p.G550R	34.00	1.71	7	glucose/sorbose dehydrogenase
	PPP2R1B	Protein Phosphatase 2 Scaffold Subunit Abeta	11_111636995_C_T	ENST00000311129.5:exon1:c.G91A:p.E31K	35.00	0.44	7	cell growth and division
	MFAP5	Microfibril Associated Protein 5	12_8803182_C_T	ENST00000359478.2:exon8:c.G251A:p.C84Y	27.80	0.21	7	extracellular matrix organization; hematopoiesis
	TCAIM	T Cell Activation Inhibitor, Mitochondrial	3_44402954_A_T	ENST00000342649.4:exon4:c.A263T:p.Y88F	24.10	0.34	6	T-cell apoptosis
	FAM49A	Family With Sequence Similarity 49 Member A; CYRIA CYFIP Related Rac1 Interactor A	2_16742741_G_A	ENST00000381323.3:exon7:c.C496T:p.R166C	34.00	2.77	7	
**Family 20**	CHD3	Chromodomain Helicase DNA Binding Protein 3	17_7810227_C_T	ENST00000330494.7:exon30:c.C4544T:p.P1515L	26.10	7.15	10	chromatin remodeling
	ATP2B2	ATPase Plasma Membrane Ca2+ Transporting 2	3_10381937_C_T	ENST00000360273.2:exon21:c.G3226A:p.V1076I	22.90	6.36	7	Ca2+ homeostasis
	OIT3	Oncoprotein Induced Transcript 3	10_74684222_A_T	ENST00000334011.5:exon7:c.A1187T:p.D396V	24.10	0.73	10	liver development and function
	DAAM1	Dishevelled Associated Activator Of Morphogenesis 1	14_59835503_C_T	ENST00000395125.1:exon25:c.C3163T:p.R1055W	34.00	1.22	10	cell polarity; Wnt signaling
	SLC6A19	Solute Carrier Family 6 Member 19	5_1210676_C_T	ENST00000304460.10:exon3:c.C461T:p.P154L	27.60	0.37	8	amino acid transport

	MYO1G	Myosin IG	7_45006305_C_T	ENST00000258787.7:exon15:c.G1915A:p.A639T	33.00	1.83	9	immune response
	DNAH2	Dynein Axonemal Heavy Chain 2	17_7691226_A_T	ENST00000572933.1:exon43:c.A6652T:p.T2218S	24.20	2.44	10	motor protein
	DARS2	Aspartyl-TRNA Synthetase 2, Mitochondrial	1_173800731_G_T	ENST00000361951.4:exon5:c.G455T:p.C152F	27.20	0.56	8	tRNA aminoacetylation; protein biosynthesis
	ATP1B2	ATPase Na+/K+ Transporting Subunit Beta 2	17_7559161_G_A	ENST00000250111.4:exon7:c.G821A:p.R274Q	29.00	0.64	6	ion transport
	RAPGEF4	Rap Guanine Nucleotide Exchange Factor 4	2_173832028_A_G	ENST00000397081.3:exon10:c.A860G:p.Y287C	27.90	1.55	9	exocytosis
**Family 21**	ADAT3	Adenosine Deaminase TRNA Specific 3	19_1912227_G_T	ENST00000329478.2:exon2:c.G181T:p.A61S	24.70	1.42	7	tRNA processing
	MORN4	MORN Repeat Containing 4	10_99379394_C_A	ENST00000307450.6:exon2:c.G17T:p.G6V	34.00	0.22	8	response to axon injury
	DDX59	DEAD-Box Helicase 59	1_200619764_A_G	ENST00000331314.6:exon5:c.T1103C:p.L368P	29.50	0.08	6	RNA metabolism
	USP28	Ubiquitin Specific Peptidase 28	11_113683078_C_A	ENST00000003302.4:exon16:c.G1892T:p.R631I	34.00	0.17	7	DNA damage-induced apoptosis
	GALNT10	Polypeptide N-Acetylgalactosaminyltransferase 10	5_153783769_C_T	ENST00000297107.6:exon8:c.C1162T:p.R388W	35.00	1.75	9	synthesis of mucin-type oligosccharides
	ABTB2	Ankyrin Repeat And BTB Domain Containing 2	11_34378544_C_A	ENST00000435224.2:exon1:c.G587T:p.G196V	28.40	1.16	7	hepatocyte growth
	PCDHGC5	Protocadherin Gamma Subfamily C, 5	5_140870047_C_T	ENST00000252087.1:exon1:c.C1240T:p.R414W	31.00	1.59	7	call adhesion in brain
	KLHL18	Kelch Like Family Member 18	3_47376277_T_A	ENST00000232766.5:exon6:c.T866A:p.L289H	25.30	2.74	7	ubiquitination; mitotic check-point

CHROM_POS_REF_ALT, chromosome_position_reference allele_alternative allele; CADD, Combined Annotation-Dependent Depletion; ExAC, the Exome Aggregation Consortium

*Deleteriousness of the variants were predicted using Sorting Intolerant from Tolerant (SIFT), Polymorphism Phenotyping version-2 (PolyPhen-2) HDIV (HumDiv), PolyPhen-v2 HVAR (HumVar), Log ratio test (LRT), MutationTaster, Mutation Assessor, Functional Analysis Through Hidden Markov Models (FATHMM), MetaSVM, MetaLR, Protein Variation Effect Analyzer (PROVEAN)

Supplementary Table 3 Loss-of-function variants segregating with the disease in the multiple myeloma families.

Family ID	GENE	Gene name	CHROM_POS_REF_ALT	VARIANT CLASSIFICATION	Ensembl transcript:exon:nucleotide: amino acid or Ensembl transcript; HGVS*	CADD	Impact on protein (MutPred-LOF; Human Splicing finder)**	Function
**Family 1**	NPFFR2	Neuropeptide FF Receptor 2	4_73003756_G_A	splicing	ENST00000308744.6; HGVSp:c.635-1G>A	26.40	yes	G-protein-coupled receptor signaling; pain modulation
**Family 2**	KRI1	KRI1 Homolog	19_10673404_C_A	splicing	ENST00000312962.6; HGVSp:c.401+1G>T	25.40	yes	
**Family 6**	FUK	Fucose Kinase	16_70500783_A_G	splicing	ENST00000288078.6; HGVSp:c.412-2A>G	23.40	yes	glycoprotein and glycolipid synthesis
	U2AF1L4	U2 Small Nuclear RNA Auxiliary Factor 1 Like 4	19_36234717_TG_T	frameshift deletion	ENST00000378975.3:exon5:c.453delC:p.P151fs	24.20	0.29	mRNA processing/splicing
	AHI1	Abelson Helper Integration Site 1	6_135752371_G_C	stopgain SNV	ENST00000367800.4:exon15:c.C2348G:p.S783X	38.00	0.53	cilium biogenesis/degradation
	DNLZ	DNL-Type Zinc Finger	9_139258036_C_T	stopgain SNV	ENST00000371738.3:exon1:c.G131A:p.W44X	35.00	0.33	chaperone
	MYO19	Myosin XIX	17_34867295_C_G	splicing	ENST00000614623.4; HGVSp:c.897-1C>G	25.00	yes	motor protein
	MUC17	Mucin 17, Cell Surface Associated	7_100679568_CA_C	frameshift deletion	ENST00000306151.4:exon3:c.4872delA:p.S1624fs	23.30	0.4	homeostasis of mucosal surfaces
	IARS1	Isoleucyl-tRNA Synthetase 1	9_95004514_T_TATGA	frameshift insertion	ENST00000375643.3:exon29:c.3097_3098insTCAT:p.I1033fs	35.00	0.42	aminoacyl-tRNA synthetase
	CORIN	Corin, Serine Peptidase	4_47667210_A_C	stopgain SNV	ENST00000273857.4:exon11:c.T1428G:p.Y476X	36.00	0.62	serine protease
	LONP2	Lon Peptidase 2, Peroxisomal	16_48311302_TG_T	frameshift deletion	ENST00000285737.4:exon8:c.1296delG:p.V432fs	22.60	0.68	peroxisome homeostasis
**Family 7**	IFT74	Intraflagellar Transport 74	9_26990150_C_T	stopgain SNV	ENST00000443698.1:exon8:c.C544T:p.R182X	41.00	0.43	cilium biogenesis/degradation
	ELOVL2	ELOVL Fatty Acid Elongase 2	6_10990562_G_A	stopgain SNV	ENST00000354666.3:exon6:c.C619T:p.Q207X	40.00	0.56	fatty acid biosynthesis

	RESP18	Regulated Endocrine Specific Protein 18	2_220197293_AG_A	frameshift deletion	ENST00000333527.5:exon2:c.184delC:p.L62fs	26.00	0.27	regulatory role in corticotrophs
	CDH19	Cadherin 19	18_64202276_AG_A	frameshift deletion	ENST00000262150.2:exon8:c.1282delC:p.L428fs	25.70	0.47	cell adhesion
**Family 8**	CENPO	Centromere Protein O	2_25038626_G_GT	splicing	ENST00000380834.2; HGVSp:c.594+2dupT	25.30	no	mitotic check-point
**Family 10**	IL3RA	Interleukin 3 Receptor Subunit Alpha	X_1471373_A_AT	frameshift insertion	ENST00000331035.4:exon6:c.591dupT:p.D197fs	22.70	0.44	immune response
	IL17REL	Interleukin 17 Receptor E Like	22_50436595_G_A	stopgain SNV	ENST00000389983.2:exon10:c.C745T:p.Q249X	27.90	0.37	interleukin 17 rececptor activity
**Family 11**	CRYL1	Crystallin Lambda 1	13_21086676_G_A	stopgain SNV	ENST00000298248.7:exon2:c.C55T:p.R19X	37.00		glucose catabolism
	ARHGAP40	Rho GTPase Activating Protein 40	20_37270452_G_A	splicing	ENST00000373345.4; HGVSp:c.1203+1G>A	24.20	no	GTPase activation
	ABCC11	ATP Binding Cassette Subfamily C Member 11	16_48212531_C_A	stopgain SNV	ENST00000394747.1:exon23:c.G3325T:p.E1109X	37.00	0.39	transport of small molecules/multidrug resistance
**Family 13**	GHSR	Growth Hormone Secretagogue Receptor	3_172165893_C_T	stopgain SNV	ENST00000241256.2:exon1:c.G311A:p.W104X	36.00	0.45	G-protein coupled receptor; growth hormone secretion
	SLC30A5	Solute Carrier Family 30 Member 5	5_68396699_CAT_C	frameshift deletion	ENST00000396591.3:exon2:c.150_151del:p.50_51del	35.00	0.55	zink transport
	ARHGEF19	Rho Guanine Nucleotide Exchange Factor 19	1_16534543_G_T	stopgain SNV	ENST00000270747.3:exon3:c.C590A:p.S197X	37.00	0.44	GTPase activation
	CLSPN	Claspin	1_36226083_G_T	stopgain SNV	ENST00000318121.3:exon8:c.C1439A:p.S480X	34.00	0.44	cell cycle regulation
**Family 15**	NBAS	NBAS Subunit Of NRZ Tethering Complex	2_15555780_C_A	stopgain SNV	ENST00000281513.5:exon25:c.G2827T:p.E943X	43.00	0.49	Golgi to endoplasmic reticulum transport
	CSGALNACT2	Chondroitin Sulfate N-Acetylgalactosaminyltransferase 2	10_43659357_G_T	stopgain SNV	ENST00000374466.3:exon5:c.G1024T:p.E342X	47.00	0.54	chondroitin sulfate synthesis
**Family 16**	CNGA1	Cyclic Nucleotide Gated Channel Subunit Alpha 1	4_47939805_G_GT	frameshift insertion	ENST00000514170.1:exon11:c.704dupA:p.K235fs	25.90	0.49	phototransduction
**Family 17**	DPYD	Dihydropyrimidine Dehydrogenase	1_98205966_GATGA_G	frameshift deletion	ENST00000370192.3:exon4:c.299_302del:p.100_101del	36.00	0.39	pyrimidine catabolism

**Family 19**	TMPRSS15	Transmembrane Serine Protease 15	21_19715821_A_C	splicing	ENST00000284885.3; HGVSp:c.1428+2T>G	24.00		activation of pancreatic proteolytic proenzymes
**Family 20**	COL19A1	Collagen Type XIX Alpha 1 Chain	6_70854808_A_G	splicing	ENST00000322773.4; HGVSp:c.1681-2A>G	23.20	yes	cell adhesion
	POP5	POP5 Homolog, Ribonuclease P/MRP Subunit	12_121018949_GA_G	frameshift deletion	ENST00000357500.4:exon2:c.131delT:p.F44fs	33.00	0.35	rRNA/tRNA processing
	HMGCLL1	3-Hydroxymethyl-3-Methylglutaryl-CoA Lyase Like 1	6_55406595_G_A	stopgain SNV	ENST00000398661.2:exon4:c.C319T:p.R107X	36.00	0.57	ketogenesis
	CARF	Calcium Responsive Transcription Factor	2_203842036_GA_G	frameshift deletion	ENST00000402905.3:exon13:c.1540delA:p.T514fs	33.00	0.35	transcription regulation
	CATSPER3	Cation Channel Sperm Associated 3	5_134345082_C_T	stopgain SNV	ENST00000282611.6:exon6:c.C838T:p.R280X	35.00	0.52	voltage-gated calcium channel
	SHPK	Sedoheptulokinase	17_3527481_G_A	stopgain SNV	ENST00000225519.3:exon3:c.C355T:p.R119X	23.80		glucose metabolism

CHROM_POS_REF_ALT, chromosome_position_reference allele_alternative allele; CADD, Combined Annotation-Dependent Depletion; LOF, loss-of-function;
* For LOF variants (frameshift and stopgain), Ensembl transcript:exon:nucleotide change:amino acid change are shown. For splice site variants Ensemble transcript; Human Genome Variation Society (HGVS) sequence variant nomenclature is used.
** For LOF variants (frameshift and stopgain), pathogenic and neutral variants were predicted using MutPred-LOF (http://mutpredlof.cs.indiana.edu/index.html) with a threshold score of 0.50 at 5% false positive rate. Human Splicing Finder (http://www.umd.be/HSF/HSF.shtml) was used to evaluate the effect of splice site variants, with yes/no score.

Supplementary Table 4. Copy number variants segregating with the disease in multiple myeloma families.

Family ID	CHROM_START_END	Type (size)	GENE	Gene name	Function
Family 5	4_15936942_16178663	DEL (241.72 kb)	FGFBP1	Fibroblast Growth Factor Binding Protein 1	cell proliferation, differentiation and migration
			FGFBP2	Fibroblast Growth Factor Binding Protein 2	cytotoxic lymphocyte-mediated immunity
			PROM1	Prominin 1	cell differentiation, proliferation and apoptosis
			TAPT1	Transmembrane Anterior Posterior Transformation 1	primary cilia formation, osteogenesis
	22_30889887_30892208	DUP (2.32 kb)	SEC14L4	SEC14 Like Lipid Binding 4	transport of hydrophobic ligands
Family 7	3_43641626_43647605	DEL (5.98 kb)	ANO10	Anoctamin 10	calcium-activated chloride channel
	12_50363908_50364605	DEL (0.68kb)	AQP6	Aquaporin 6	kidney-specific water channel
Family 10	1_151028877_151033279	DUP (4.40 kb)	MLLT11	MLLT11 Transcription Factor 7 Cofactor	regulation of lymphoid development
			CDC42SE1	CDC42 Small Effector 1	organization of the actin cytoskeleton
Family 11	10_126172458_151033279	DEL (0.69kb)	LHPP	Phospholysine Phosphohistidine Inorganic Pyrophosphate Phosphatase	phopshatase
Family 17	1_87029094_87039091	DEL (10.00 kb)	CLCA4	Chloride Channel Accessory 4	calcium-activated chloride conductance

DEL, deletion; DUP, duplication

Supplementary Figure 1. Pedigrees of the multiple myeloma families. For cancer patients age of diagnosis is shown, for healthy family members age at sampling. Families 18-21 consisted of two first- or second-degree relatives diagnosed with MM and are not shown.

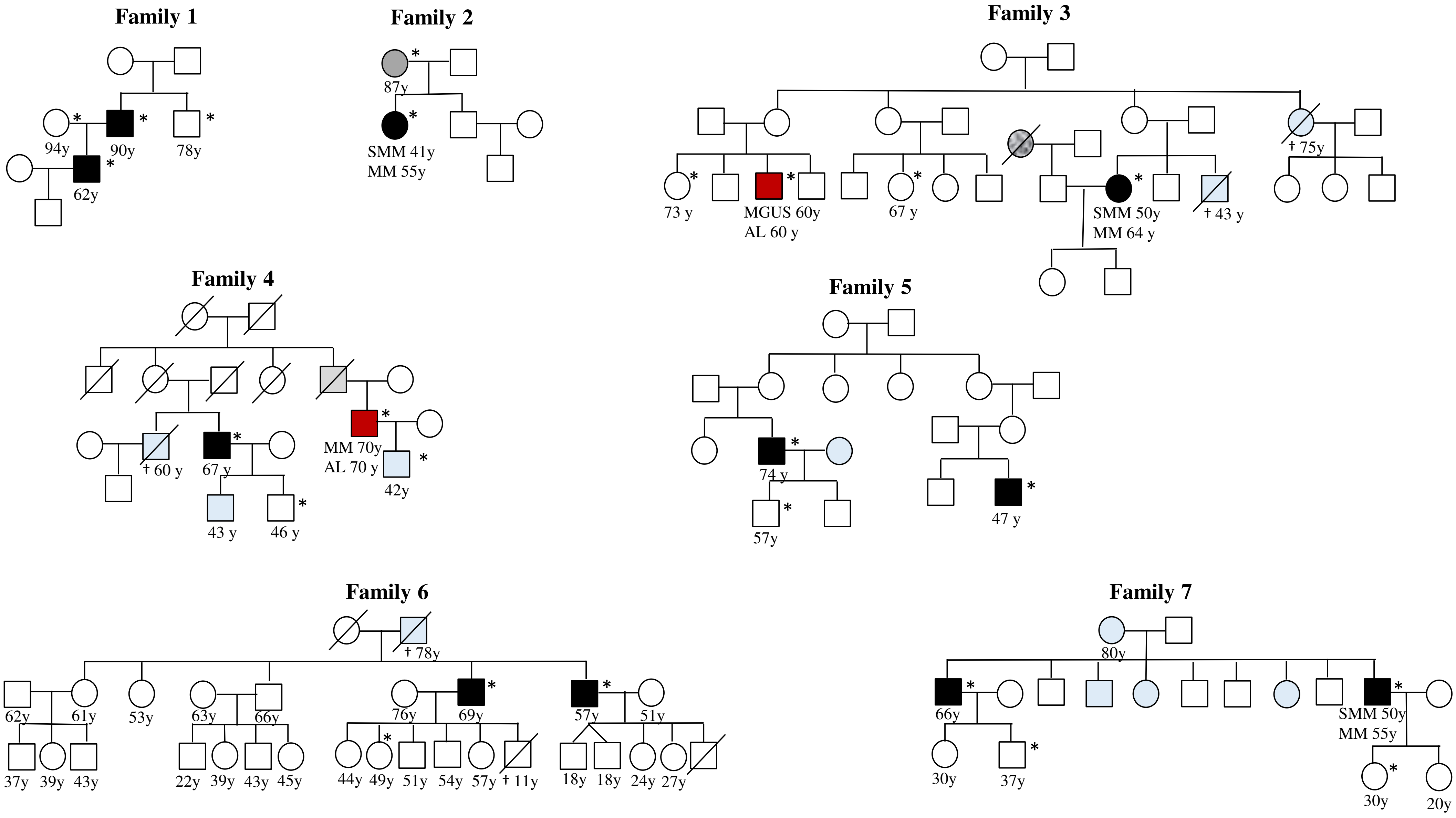

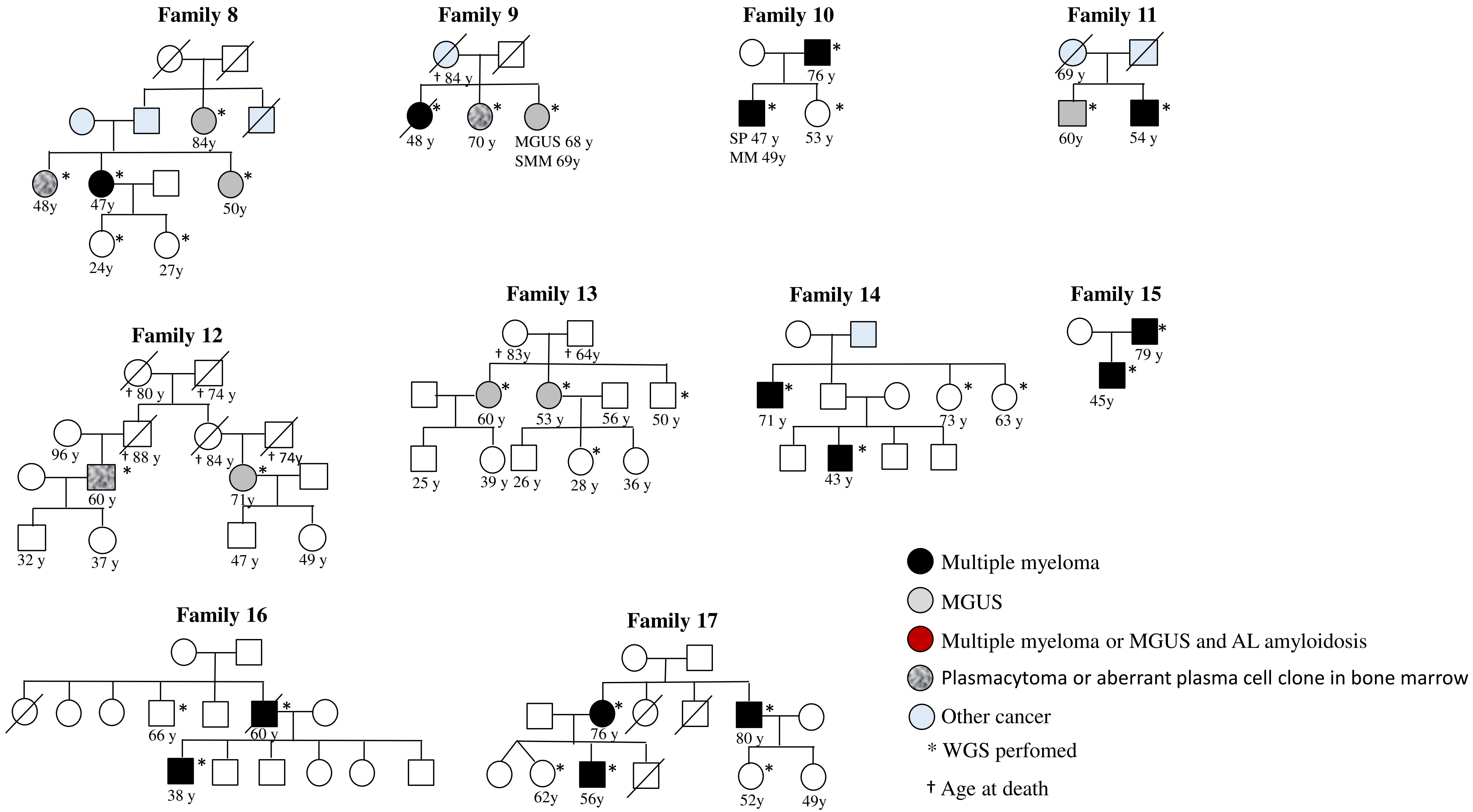

Family 8
84y
48y
47y
50y
24y
27y
Family 9
† 84 y
48 y
70 y
MGUS 68 y
SMM 69y
Family 10
76 y
SP 47 y
MM 49y
53 y
Family 11
69 y
60y
54 y
Family 12
† 80 y
† 74 y
96 y
† 88 y
† 84 y
† 74y
60 y
71y
32 y
37 y
47 y
49 y
Family 13
† 83y
† 64y
60 y
53 y
56 y
50 y
25 y
39 y
26 y
28 y
36 y
Family 14
71 y
73 y
63 y
43 y
Family 15
79 y
45y
Family 16
66 y
60 y
38 y
Family 17
76 y
80 y
62y
56y
52y
49y
Multiple myeloma
MGUS
Multiple myeloma or MGUS and AL amyloidosis
Plasmacytoma or aberrant plasma cell clone in bone marrow
Other cancer
* WGS perfomed
† Age at death
